# Statistical Modeling of Near-Surface Aggregate Size Distributions in Concrete

**DOI:** 10.3390/ma19071395

**Published:** 2026-03-31

**Authors:** Alexander Haynack, Thomas Kränkel, Christoph Gehlen, Jithender J. Timothy

**Affiliations:** Centre for Building Materials (CBM), Chair of Materials Science and Testing, Department of Materials Engineering, TUM School of Engineering and Design, Technical University of Munich, 85748 Garching b. Munich, Germany; thomas.kraenkel@tum.de (T.K.); gehlen@tum.de (C.G.); jithender.timothy@tum.de (J.J.T.)

**Keywords:** aggregate distribution optimization, concrete, mesoscale modeling, wall effect

## Abstract

This study presents a distribution-optimized mesostructure estimation method for statistically modeling near-surface aggregate size distributions in concrete by optimizing the spatial arrangement of polydisperse spherical aggregates with respect to formwork boundaries. The approach is based on minimizing the deviation between a generated cumulative aggregate volume function and an idealized linear target function corresponding to a constant area fraction along the specimen depth. To enable efficient computation for systems containing a large number of aggregates, grain size groups derived from the grading curve are represented using symmetric Beta distributions, allowing each group to be described by a single shape parameter. The resulting optimization problem is solved using a derivative-free Powell algorithm. The method inherently captures wall effects, leading to a migration of smaller aggregates toward the specimen boundaries to compensate for the geometric constraints of bigger aggregates. Experimental validation was performed for a single concrete mixture and specimen geometry by determining the depth-dependent mean bulk density of a concrete cube using incremental surface grinding combined with high-resolution 3D laser scanning. The optimized mesostructure shows strong agreement with measured density profiles for the investigated specimen. While the validation is limited to a single mixture and geometry, the results indicate that the proposed method is a computationally efficient approach for incorporating wall effects into mesoscale concrete models. Furthermore, increasing aggregate volume fractions intensify the near-surface accumulation of fine particles.

## 1. Introduction

Concrete is a composite material fundamentally consisting of cement, mixing water, fine aggregates (sand), and coarse aggregates (gravel). Upon hardening, the hydration products form a solid matrix surrounding the aggregates. On a mesoscopic scale ($10^{-4}$ m < *l* < $10^{-1}$ m) [1], the heterogeneous mixture can be simplified as a two-phase system, consisting of a cementitious matrix and aggregates. The positions of the polydisperse aggregates are constrained by the size of the aggregates and the physical boundaries of the formwork. In addition, between the aggregates and the cementitious matrix, interfacial transition zones (ITZ) are formed [2,3,4,5].

The generation of realistic virtual concrete mesostructures is a significant area of research, driven by the need for accurate input in numerical simulations to predict concrete behavior [6,7]. These models aim to statistically replicate the configuration, size, and distribution of aggregates within the cementitious matrix [8]. The literature describes a variety of different methods of generating numerical mesoscale models. A comprehensive review was given by Ren [8]. A common approach is described as the take-and-place method. In this method, spherical or ellipsoidal aggregates, sampled from a known grading curve, are randomly placed inside the boundaries of a virtual specimen, ensuring no overlap with previously placed aggregates [9]. Furthermore, methods have been explored to generate more realistic aggregate geometries, moving from simple spheres or ellipsoids to polyhedral shapes, with the ability to consider features like irregular and concave faces [6,10,11]. Other approaches include techniques based on Voronoi tessellation and Delaunay triangulation to control particle shape and size in the system [12,13,14]. Some models also consider the ITZ between aggregates and mortar, which is known to be a critical area for crack initiation and concrete durability [15]. In addition, current research explores methods to consider non-uniform aggregate distributions due to segregation, which can significantly impact concrete properties [7]. The particle shape has a significant impact on the mechanical and rheological behavior of granular composite systems [16]. Furthermore, molecular dynamics simulations have been used to analyze the influence of ionic substitutions on the structure and properties of cementitious gels, providing atomistic insights into hydration mechanisms [17].

**Figure 1:**
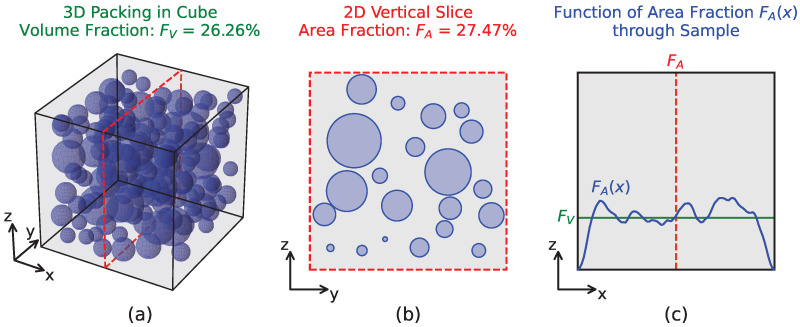
(**a**) 3D packing of spheres inside cube; (**b**) 2D vertical slice of cube with sphere circle segments; (**c**) function of area fraction through sample.

Figure 1a exemplarily shows a random packing of polydisperse spheres within a cube. The volume fraction $F_{V}$ describes the ratio between the total volume of the boundary geometry and the volume of all spheres. A vertical slice through the cube at any position, as depicted in Figure 1b, yields the area fraction $F_{A}$, describing the ratio between the total area of the sliced boundary geometry and the area of all intersected sphere segments. The function of area fraction $F_{A}\left(x\right)$ describes the progression of $F_{A}$ along the axis perpendicular to the vertical slices, see Figure 1c. It can be seen that $F_{A}\left(x\right)$ is never constant and exhibits values meandering around $F_{V}$.

In this study, a novel methodology is presented, where concrete mesostructures are generated by minimizing the difference between the actual and idealized homogeneous cumulative volume fraction functions $F_{V}\left(x\right)$ of a polydisperse distribution of spherical aggregates. The functions for $F_{V}\left(x\right)$ have been derived from integrating $F_{A}\left(x\right)$ over one axis of the geometry, see Equation (1).

(1) $$F_{V}\left(x\right)=\int_{0}^{x}F_{A}\left(x\right)\;dx\;$$

The aggregates in the system are assembled based on the assumption of a constant $F_{A}\left(x\right)$ and thus linear $F_{V}\left(x\right)$ within the geometry, which results in smaller particles migrating towards the near-surface region of the sample as a consequence of the optimization process. It is important to note that the method presented in this study does not reconstruct explicit three-dimensional aggregate positions or the contact topology of aggregates. Instead, it provides a statistically consistent mesostructure representation along a specific axis direction (depth) of the specimen, which is particularly suited for studies of boundary-induced heterogeneity and density variation. For mechanical simulations, i.e., stress transfer, ITZ connectivity, or crack initiation, explicit mesostructure models are recommended. The focus of this method is on the representation of the border zones of the concrete laboratory specimens (wall effect) [18].

Existing wall effect modeling approaches are typically based on explicit particle packing simulations or geometric placement algorithms, in which boundary effects arise from direct particle-to-particle or particle-to-wall interactions [11,19,20]. While these approaches provide detailed spatial representations, they require high computational effort. As reported in prior work, packing algorithms become increasingly inefficient at high volume fractions [6]. In contrast, the proposed distribution-optimized mesostructure estimation method formulates the problem as an inverse cumulative-volume matching task. Instead of explicitly simulating contact mechanics, the method enforces a target cumulative volume function along the specimen depth and determines depth-dependent aggregate distributions through optimization. This formulation reduces computational complexity while preserving the geometric characteristics of boundary-induced redistribution.

A Python (version 3.12.12) implementation of the methodology is available online: https://github.com/ahaynack/DOME (accessed on 11 March 2026).

## 2. Mesostructure Generation

**Figure 2:**
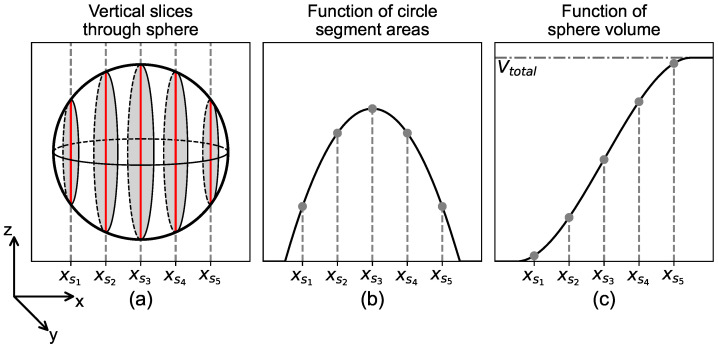
(**a**) Slices; (**b**) areas; (**c**) volume of sphere.

The first fundamental concept of the presented method is the representation of the volume of a spherical aggregate as the cumulative sum of its intersected circle segment areas along an axis, i.e., the integral of its area. Figure 2a depicts a spherical aggregate, which is intersected by vertical slices. At the locations of the slices, a circle segment area with radius $r_{s_{i}}$ is formed. The function of the circle segment areas $A_{s_{i}}\left(x\right)$ is shown in Figure 2b. Integrating $A_{s_{i}}\left(x\right)$ ultimately results in the function of sphere volume $V_{s_{i}}\left(x\right)$, as depicted in Figure 2c.

**Figure 3:**
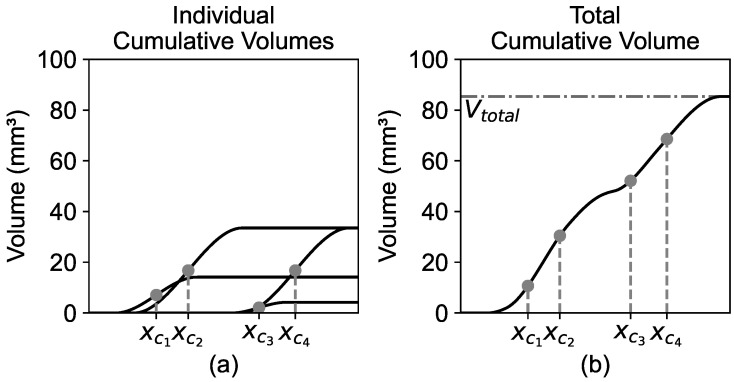
(**a**) Individual cumulative volumes; (**b**) total cumulative volume of spheres.

The shape of the volume function of each aggregate *i* is governed by the radius $r_{i}$ and the x-position of the center of the aggregate $x_{c_{i}}$. Figure 3a shows four individual volume functions, $V_{i}\left(x\right)$, of spheres with different radii and random positions along the x-axis. In Figure 3b, the total volume function $V_{\mathrm{tot}}\left(x\right)$ is formed from the individual volume functions.

The mathematical formulation of $V_{i}\left(x\right)$ is shown in Equation (2):

(2) $$V_{i}\left(x\right)=\left\{\begin{array}{cc} 0 & x\leq x_{c_{i}}-r_{i}\\ \pi \left(r_{i}^{2}\left(x-x_{c_{i}}\right)-\frac{{\left(x-x_{c_{i}}\right)}^{3}}{3}+\frac{2}{3}r_{i}^{3}\right) & x_{c_{i}}-r_{i}<x<x_{c_{i}}+r_{i}\\ \frac{4}{3}\pi r_{i}^{3} & x\geq x_{c_{i}}+r_{i} \end{array}\right.$$

where $V_{i}\left(x\right)$ denotes the cumulative volume function of sphere *i* along the *x*-axis, $r_{i}$ is the radius of sphere *i*, and $x_{c_{i}}$ is the *x*-coordinate of its center. The total volume function $V_{\mathrm{tot}}\left(x\right)$ is calculated according to Equation (3):

(3) $$V_{\mathrm{tot}}\left(x\right)=\sum_{i=1}^{n}V_{i}\left(x\right)$$

where $V_{i}\left(x\right)$ denotes the cumulative volume function of sphere *i* along the *x*-axis, $r_{i}$ is the radius of sphere *i*, $x_{c_{i}}$ is the *x*-coordinate of its center, and *n* is the number of spheres.

**Figure 4:**
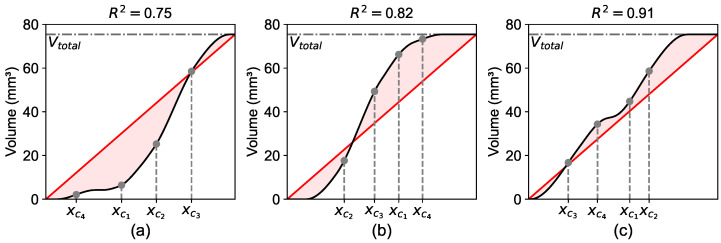
$R^{2}$ score comparisons of different spatial configurations of spheres. (**a**) $R^{2}=0.75$; (**b**) $R^{2}=0.82$; (**c**) $R^{2}=0.91$. Black solid lines show the total volume functions $V_{\mathrm{tot}}\left(x\right)$ based on the spatial configurations of spheres. Red solid lines display the respective linear volume functions $V_{\mathrm{lin}}\left(x\right)$. Gray dashed lines indicate the individual sphere centers.

The second fundamental concept of the presented method is the comparison of the total volume function $V_{\mathrm{tot}}\left(x\right)$ with an idealized homogeneous volume function $V_{\mathrm{lin}}\left(x\right)$. This linear cumulative target function, $V_{\mathrm{lin}}\left(x\right)$, represents the reference case of a statistically homogeneous aggregate distribution in the bulk material. It describes the integral of constant area fractions across the x-axis, resulting in a linear volume function. Deviations from this condition arise primarily due to geometric boundary effects. Minimizing the differences between these two volume functions by rearranging the positions of the individual sphere centers $x_{c_{i}}$ results in an even distribution of the actual spheres in the system, constrained by the physical boundaries of the sample geometry. Therefore, the optimization seeks the particle distribution that accounts for the geometric constraints near the specimen boundaries with the assumption of a homogeneous bulk composition. Figure 4 shows the total volume functions $V_{\mathrm{tot}}\left(x\right)$ for three different spatial configurations of spheres within the system. Each function is compared to their linear volume function $V_{\mathrm{lin}}\left(x\right)$ by calculating the coefficient of determination $R^{2}$, defined in Equation (4).

(4) $$R^{2}=1-\frac{SS_{\mathrm{res}}}{SS_{\mathrm{tot}}}$$

The term $SS_{\mathrm{res}}$ represents the residual sum of squares and is calculated as shown in Equation (5):

(5) $$SS_{\mathrm{res}}=\sum_{i=1}^{n}{\left(y_{i}-{\hat{y}}_{i}\right)}^{2}$$

The term $SS_{\mathrm{tot}}$ represents the total sum of squares, proportional to the variance of the observed data, defined in Equation (6):

(6) $$SS_{\mathrm{tot}}=\sum_{i=1}^{n}{\left(y_{i}-\bar{y}\right)}^{2}$$

where

- $R^{2}$: the coefficient of determination;
- $y_{i}$: the *y*-value of the *i*-th point in the observed data;
- ${\hat{y}}_{i}$: the corresponding *y*-value on the fitted linear line (the predicted value);
- $\bar{y}$: the mean of the observed *y*-values, calculated as $\frac{1}{n}\sum_{i=1}^{n}y_{i}$;
- *n*: the total number of data points.

While the explicit rearrangement of individual sphere positions works well for a small number of spheres, simulations based on actual grading curves for concrete or mortar samples, potentially containing millions of aggregates, can become computationally prohibitive. To address this, the polydisperse grading curve is divided into distinct grain size groups. For each group, the spatial arrangement (depth) of the aggregates is modeled using a symmetrical Beta distribution, defined in Equation (7). This approach relies on the assumption that the boundary conditions are identical at both ends of the sample. By setting the two standard shape parameters equal to each other (β=α), the distribution becomes symmetric around x=0.5, controlled by a single parameter α. Since the standard Beta distribution is defined on the interval [0,1], the physical positions are scaled by the total sample length.

(7) $$B\left(x,\alpha \right)=\frac{\Gamma \left(2\alpha \right)x^{\alpha -1}{\left(1-x\right)}^{\alpha -1}}{\Gamma {\left(\alpha \right)}^{2}}$$

where

- B(x,α): The probability density indicating the likelihood of an aggregate from a specific grain size group being located at normalized depth *x*.
- *x*: The normalized depth of the aggregate, defined as x=d/L, where *d* is the physical depth and *L* is the total length of the sample (0≤d≤L).
- α: The shape parameter for the specific grain size group (α>0). Due to the symmetry assumption (β=α), this parameter controls the concentration of aggregates relative to the following boundaries:
  − If α>1, aggregates concentrate toward the center of the sample (bell-shaped distribution);
  − If α<1, aggregates concentrate toward the border zones (U-shaped distribution);
  − If α=1, aggregates are uniformly distributed along the length *L*.
- Γ(·): The Gamma function.

**Figure 5:**
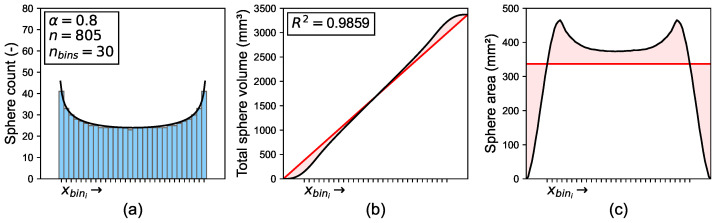
(**a**) Histogram of sphere centers (blue bars) based on symmetrical Beta distribution (black line); (**b**) total sphere volume function (black line) compared to linear volume function (red line); (**c**) sphere area function (black line) compared to constant area function (red line). Positions of individual sphere centers indicated by $x_{{\mathrm{bin}}_{i}}$.

Analogously to the individual aggregates, the distributions of aggregates result in the total volume function $V_{\mathrm{tot}}\left(x\right)$, which can be compared to the linear volume function $V_{\mathrm{lin}}\left(x\right)$. Figure 5a exemplarily shows the distribution of *n* = 805 spheres of the same radius. The sphere positions are segmented into $n_{\mathrm{bins}}$ = 30 bins along the x-axis. These exemplary values were chosen to account for an adequate visual clarity. The magnitudes of sphere bins correspond to a symmetrical Beta distribution, which can be described solely with parameter α. The $R^{2}$ score of the spheres’ total volume function $V_{\mathrm{tot}}\left(x\right)$, generated from the arrangement of sphere bins, and the linear volume function $V_{\mathrm{lin}}\left(x\right)$ are depicted in Figure 5b. Afterwards, both volume functions can be derived, resulting in the area functions along the x-axis, see Figure 5c.

For a given grain size distribution, the grouping of aggregates into grain size groups is defined. By default, the groups follow standard sieve mesh sizes of the grading curve (e.g., 0.125, 0.25, 0.5, 1, 2, 4, 8, 16 mm). Alternatively, a finer discretization of the grain size range can be applied, resulting in a larger number of grain size groups. Each grain size group is represented by an individual shape parameter $\alpha_{i}$, hence increasing the number of groups directly increases the number of optimization variables. Consequently, a finer grouping improves the resolution of the grading representation but also increases the dimensionality and computational effort of the optimization problem.

**Figure 6:**
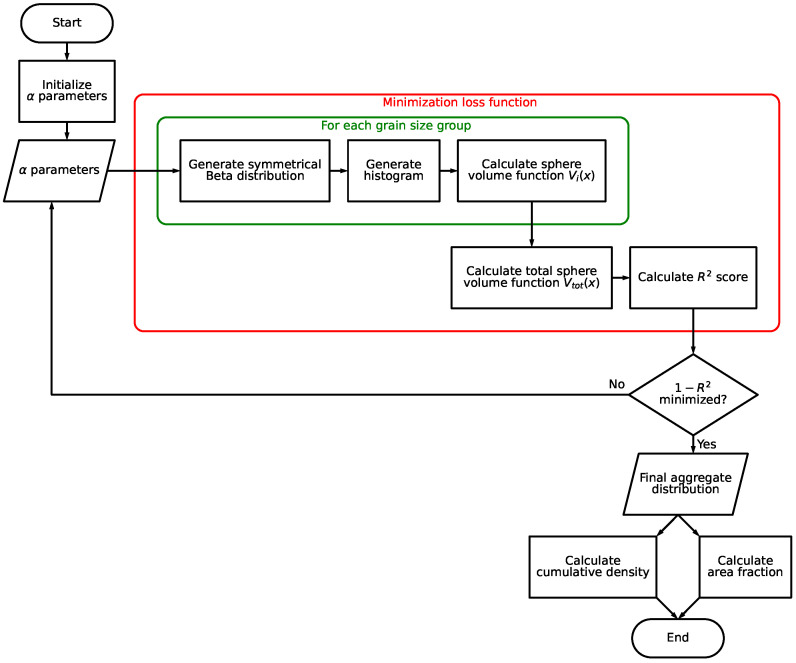
Flowchart of the main optimization algorithm.

Figure 6 displays a flowchart of the main optimization algorithm for the generation of the mesostructure. At first, an initial set of α parameters is set for the initial guess. These parameters are then passed on to the minimization module. The optimization problem is solved by minimizing the loss function, which consists of five sub-modules. For each grain size group *i*, a histogram of spheres along the x-axis is generated based on the parameter of the symmetrical Beta distribution $\alpha_{i}$. Subsequently, the respective sphere volume function $V_{i}\left(x\right)$ is generated. Afterwards, the sum of each grain size group’s sphere total volume function $V_{\mathrm{tot}}\left(x\right)$, which is compared to the linear volume function $V_{\mathrm{lin}}\left(x\right)$, is calculated according to Equation (8):

(8) $$V_{\mathrm{tot}}\left(x\right)=\sum_{i=1}^{n}V_{i}\left(x\right)$$

where *n* denotes to total number of grain size groups. The resulting $R^{2}$ score is being minimized according to the loss function L(α), as depicted in Equation (9).

(9) $$L\left(\alpha \right)=1-R^{2}\left(\alpha \right)$$

where $R^{2}\left(\alpha \right)$ denotes the coefficient of determination between the target $V_{\mathrm{lin}}\left(x\right)$ and generated $V_{\mathrm{tot}}\left(x\right)$ values. The formulation of L(α) ensures that the loss decreases as the fit improves, i.e., as $R^{2}\left(\alpha \right)\to 1$, the loss L(α)→0.

Calculating the aggregate histograms involves rounding continuous distributions to integer sphere counts. This creates a stepwise function where small changes in the shape parameters α may not change the sphere count, causing standard gradient-based optimization methods to fail (local gradient is zero) [21]. To overcome this, the optimization is performed using Powell’s method [22]. This is a derivative-free optimization algorithm, which, instead of calculating a slope, minimizes the loss by searching along specific directions one by one (sequential line searches). The shape parameters α are updated iteratively according to Equation (10) [23]:

(10) $$\alpha_{\mathrm{new}}=\alpha_{\mathrm{old}}+\lambda d$$

where

- $\alpha_{\mathrm{old}}/\alpha_{\mathrm{new}}$: The vector containing the shape parameters for all grain size groups (i.e., $\alpha =[\alpha_{1},\alpha_{2},\ldots ,\alpha_{n}]$).
- d: The search direction vector. In the initial steps, this vector isolates individual grain size groups (i.e., varying one parameter $\alpha_{i}$ while keeping the others fixed). As the algorithm progresses, d evolves into a combined direction, adjusting multiple shape parameters simultaneously.
- λ: The scalar step size that determines how far the algorithm moves the parameters along the direction d to reach the minimum loss for that specific search step.

By cycling through these line searches, the algorithm converges to the optimal aggregate distribution without requiring an analytical gradient.

Once the minimization is successful, the algorithm yields the optimized aggregate distribution according to the shape parameters α of the final step of the optimization process. Ultimately, the aggregate distribution can be used to either calculate the depth-dependent mean bulk density or calculate the depth-dependent area fraction of the system. It has to be noted that the depth-dependent mean bulk density can only be determined if the individual density values of the cementitious matrix and aggregates are known.

The proposed mesostructure generation approach minimizes the deviation between the actual and idealized cumulative aggregate volume functions. In principle, the coefficient of determination ($R^{2}$) could be evaluated over the entire specimen depth, resulting in a global optimization objective. However, the primary structural effect investigated in this study is the near-surface accumulation of aggregates and, thus, densification of the material caused by geometric boundary effects. When a purely global objective is used, localized deviations near the specimen boundaries may be obscured and thus only have a minor influence on the overall loss function because the interior region dominates the cumulative volume profile.

To increase the sensitivity of the optimization to boundary effects, the loss function can be evaluated over a limited portion of the specimen depth. This region is defined by the so-called near-surface percentage $p_{\mathrm{ns}}$, which specifies the fraction of the specimen depth included in the objective function. For example, a near-surface percentage of 100% corresponds to a global optimization, while a value of 20% restricts the evaluation of the $R^{2}$ score to the first 20% of the specimen depth. This formulation allows the optimization process to place stronger emphasis on accurately reproducing the near-boundary aggregate distribution.

In Appendix A, a sensitivity analysis of the optimization parameters was conducted. Based on the obtained results, a parameter combination of $n_{\mathrm{bins}}$ = 801 and $p_{\mathrm{ns}}$ = 20% was selected for subsequent simulations, representing a compromise between numerical accuracy, near-surface agreement, physical plausibility, and computational efficiency.

## 3. Experimental Validation

### 3.1. General

For the experimental validation of the developed method, the depth-dependent density change of a concrete sample has been determined. With the aid of a high-resolution 3D laser scanner (Creaform HandySCAN Black Elite), the volume of a concrete sample was detected. The corresponding mass was measured with a precision scale. Afterwards, the experimental results are compared with the density values derived from the generated mesostructure.

### 3.2. Materials

The mix design investigated in this study consists of an Ordinary Portland Cement (CEM I 42.5 N, Heidelberg Materials, Burglengenfeld), according to DIN EN 197-1 [24], with a w/c ratio of 0.55. The maximum aggregate size of the mixture was 8 mm (quarzitic, naturally rounded gravel, Quarzwerke Frechen). The grain size distribution can be taken from Table 1.

Table 2 shows the concrete mixture proportion used in this study. A volume fraction of aggregates, $F_{V}$, of 0.67 was chosen. The density of aggregates $\rho_{\mathrm{aggregates}}$ was determined as 2622 kg/m^3^.

The experimental determination of the depth-dependent density change of concrete was performed exemplarily on one cube with a length of 15 cm. After casting, the sample has been kept in the formwork for 1 day. Afterwards, the sample was placed in underwater storage for 6 days, followed by a storage at 20 °C and 65 % relative humidity for 21 days. The experiments were conducted at a sample age of 28 days. Prior to testing, the total sample density $\rho_{\mathrm{total}}$ was determined as 2252 kg/m^3^.

### 3.3. Methods

#### 3.3.1. Experimental Determination of Depth-Dependent Density Change of Concrete

**Figure 7:**
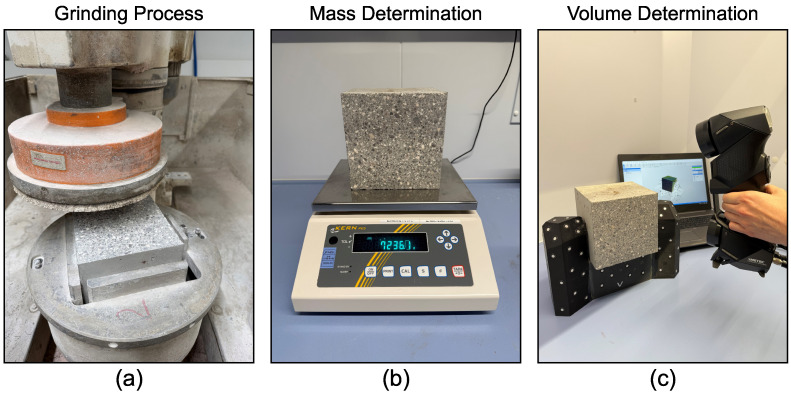
Experimental setup: (**a**) sample grinding; (**b**) mass and (**c**) volume determination of the concrete cube.

The depth-dependent density change of the sample has been determined with the aid of a high-resolution 3D laser scanner, analogously to the methodology presented in [25]. One vertical side of the concrete cube has been incrementally abraded with a grinding machine. After each grinding step, the mass and volume of the residual sample were determined. The mass was measured with a scale; the volume was measured by the 3D laser scanner. The experimental setup is displayed in Figure 7.

**Figure 8:**
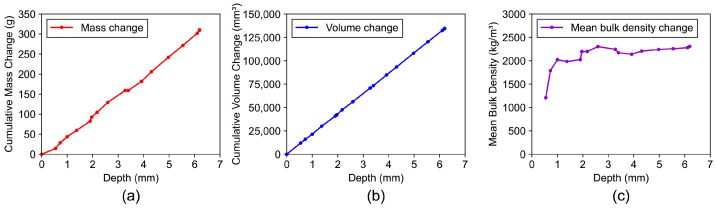
(**a**) Cumulative mass; (**b**) cumulative volume; and (**c**) mean bulk density changes of the concrete sample after incremental surface grinding.

The initial mass and volume serve as the reference values for the determination of the depth-dependent density change. For each measurement point, the change in mass and volume in respect to the reference has been determined. Afterwards, the respective change in mean bulk density was calculated by division of change in mass by change in volume, see Figure 8. The depth of each grinding step was determined by calculating the mean height loss of the abraded sample side. In total, the specimen was abraded in 17 successive layers with depth increments Δx ranging between approximately 0.25 and 0.5 mm (mean value of approximately 0.36 mm). Due to the manual adjustment of the grinding depth during the grinding procedure, the individual step heights were not uniform. The depth of each measurement point shown in Figure 8 corresponds to the cumulative height loss, while the individual step increments vary within the range of Δx.

#### 3.3.2. Generation of Mesostructure

**Figure 9:**
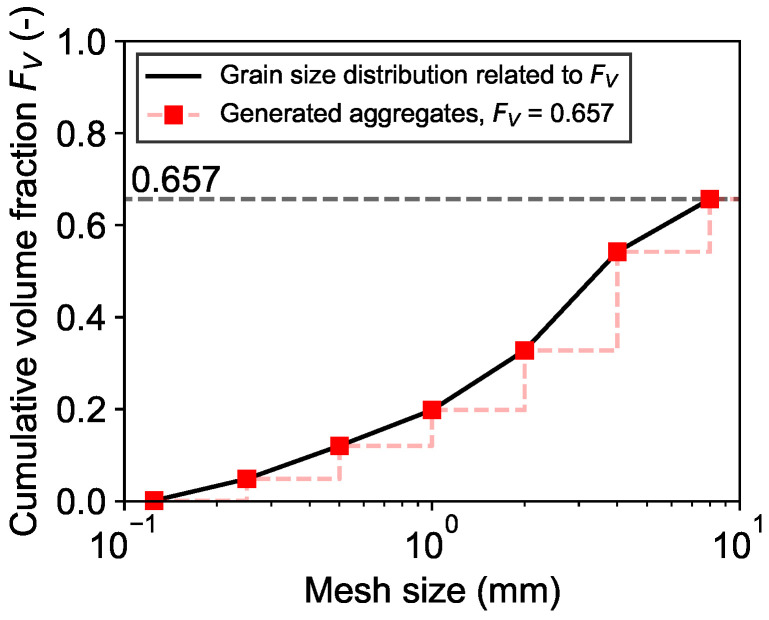
Comparison of grain size distribution related to $F_{V}$ and generated aggregates.

In preparation for the generation of the mesostructure, the aggregates in the system were generated according to their grain size distribution related to $F_{V}$, see Figure 9.

The stepwise generation of the aggregates results in the following numbers of spherical aggregates for each grain size group, see Table 3. It is important to note that no aggregates smaller than 0.125 mm or greater than 8 mm have been considered in the generation of the aggregates. These fractions were attributed as part of the volume of the cementitious matrix. As a result, the target volume fraction $F_{V}$ of 0.67 was reduced to 0.657. In addition, the grain size groups have been chosen analogously to the mesh sizes of the aggregates’ grain size distribution. Choosing a smaller mesh distance, i.e., increasing the number of grain size groups, can result in the generation of a more continuous distribution of aggregates.

**Figure 10:**
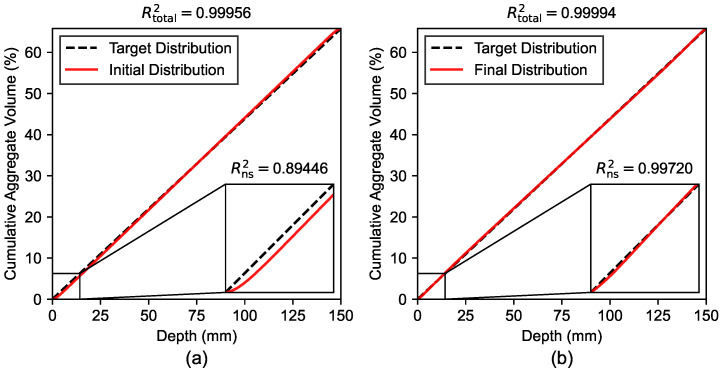
$R^{2}$ scores of (**a**) initial (non-optimized) and (**b**) final (optimized) aggregate distribution.

Figure 10 shows the $R^{2}$ scores of both the initial (non-optimized) and final (optimized) aggregate distributions compared to the linear target distribution. It can be seen that the total $R_{\mathrm{total}}^{2}$ score for both distributions yields values close to 1.0 (0.99956 and 0.99994). In contrast, with the focus on optimizing the distribution of aggregates close to the near-surface region of the sample, the coefficient of determination was also determined for the first 10% of the sample length. This $R_{\mathrm{ns}}^{2}$ of the initial distribution results in a value of 0.89446, which is a difference of approximately 11% compared to the respective $R_{\mathrm{total}}^{2}$. The final distribution yields an $R_{\mathrm{ns}}^{2}$ of 0.99720. Ultimately, the percentage difference of $R_{\mathrm{total}}^{2}$ and $R_{\mathrm{ns}}^{2}$ of the final distribution is approximately 0.27%.

**Figure 11:**
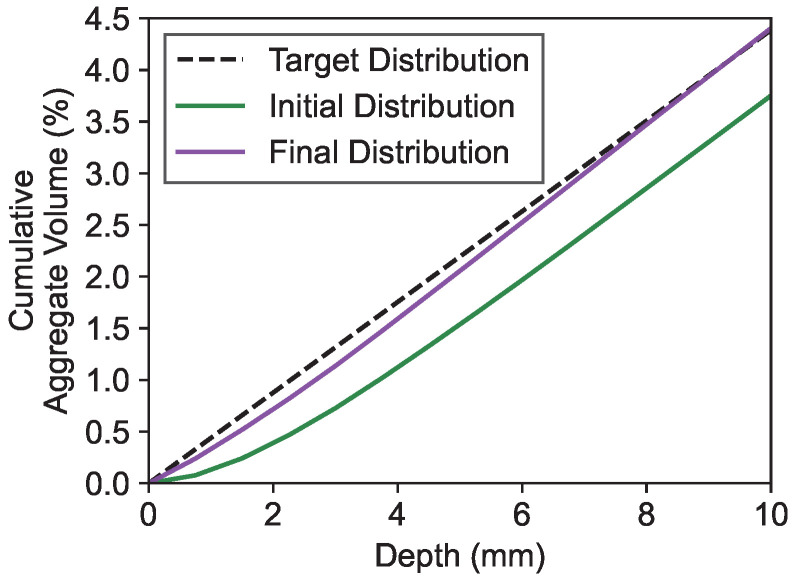
Detailed comparison of initial and final aggregate distributions in the near-surface region with linear target distribution.

Figure 11 shows a detailed view of the near-surface regions previously presented in the zoomed-in sections of Figure 10. Although the earlier magnifications yielded high $R^{2}$ scores (close to 1), suggesting an almost linear cumulative distribution, local deviations near the boundaries were not clearly visible at that scale. To better illustrate these effects, Figure 11 directly compares the initial and final distributions in the near-surface region together with the linear target distribution. This representation reveals the nonlinearity at the sample boundaries more clearly. A perfectly linear cumulative volume distribution cannot be achieved due to the boundary conditions of the mesostructure generation algorithm. The cumulative distributions represent the integrated aggregate volume along the x-axis. Due to the aggregates’ spherical shape, their contribution to the total cumulative distribution follows a characteristic sigmoidal profile, as displayed in Figure 2c, rather than a linear increase. This geometric constraint inherently causes deviations from the linear target distribution. The deviation is strongest in the initial configuration. After optimization, where smaller aggregates move towards the boundary regions, the near-surface deviation is reduced, leading to a closer approximation to linear behavior in the final distribution.

**Figure 12:**
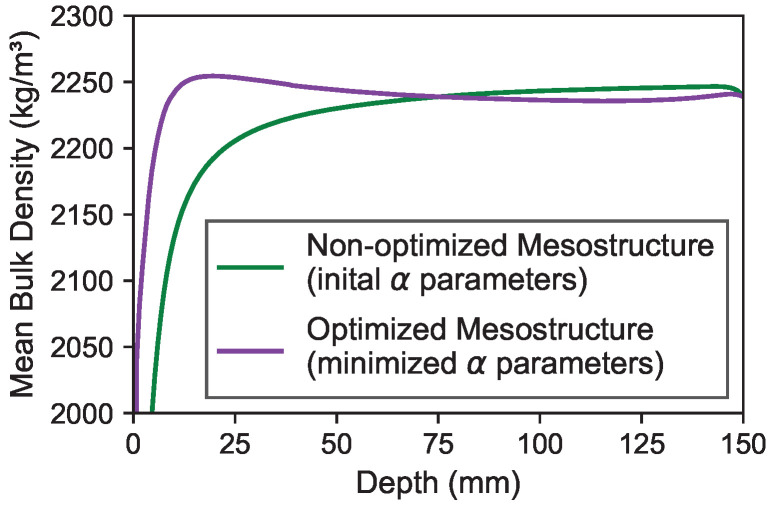
Mean bulk density of generated mesostructure.

Figure 12 depicts the depth-dependent mean bulk density values of the generated mesostructures. The green line shows the values derived from the initial α parameters. The purple line displays the density change derived from the optimized α parameters. It can be observed that both mesostructures form a pronounced depth-dependent density profile in the near-surface areas. However, due to the increased $R_{\mathrm{ns}}^{2}$ after the optimization process, the optimized mesostructure exhibits higher density values in the border zone compared to the non-optimized mesostructure.

The displayed values have been determined by Equation (11):

(11) $$\rho \left(x\right)=\frac{\rho_{\mathrm{aggregates}}V_{\mathrm{aggregates}}\left(x\right)+\rho_{\mathrm{cement}}V_{\mathrm{cement}}\left(x\right)}{V_{\mathrm{formwork}}\left(x\right)}$$

where $V_{\mathrm{aggregates}}\left(x\right)$, $V_{\mathrm{cement}}\left(x\right)$, and $V_{\mathrm{formwork}}\left(x\right)$ denote the cumulative volume functions of the aggregates, cementitious matrix, and formwork of the system. Function $V_{\mathrm{aggregates}}\left(x\right)$ was determined from the optimization algorithm. Function $V_{\mathrm{cement}}\left(x\right)$ was obtained by Equation (12):

(12) $$V_{\mathrm{cement}}\left(x\right)=V_{\mathrm{formwork}}\left(x\right)-V_{\mathrm{aggregates}}\left(x\right)$$

With the known density of aggregates $\rho_{\mathrm{aggregates}}$ (2622 kg/m^3^), total sample density $\rho_{\mathrm{total}}$ (2252 kg/m^3^), and volume fraction of aggregates $F_{V}$ (0.67), the density of the cementitious matrix $\rho_{\mathrm{cement}}$ was derived as 1502 kg/m^3^. This matrix density represents an effective density that implicitly accounts for capillary porosity, entrapped air, and unresolved microstructural heterogeneity. It should therefore be interpreted as a bulk phase parameter rather than the intrinsic density of hydrated cement paste.

**Figure 13:**
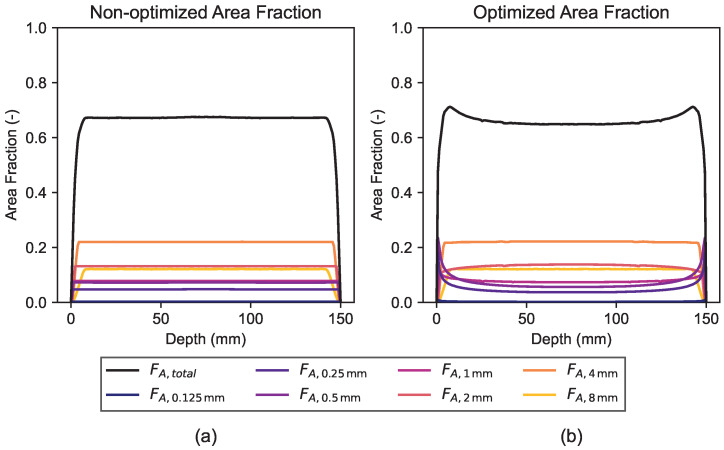
Area fraction of (**a**) non-optimized and (**b**) optimized aggregate distribution.

Figure 13 shows the depth-dependent area fractions derived from the generated mesostructures with the initial (non-optimized) and final (optimized) α parameters. Analogously to the mean bulk density change, the area fraction of the final distribution shows peaks close to the border zones of the sample, indicating a higher accumulation of (finer) aggregates compared to the initial uniform distribution.

### 3.4. Comparison of Mean Bulk Densities

**Figure 14:**
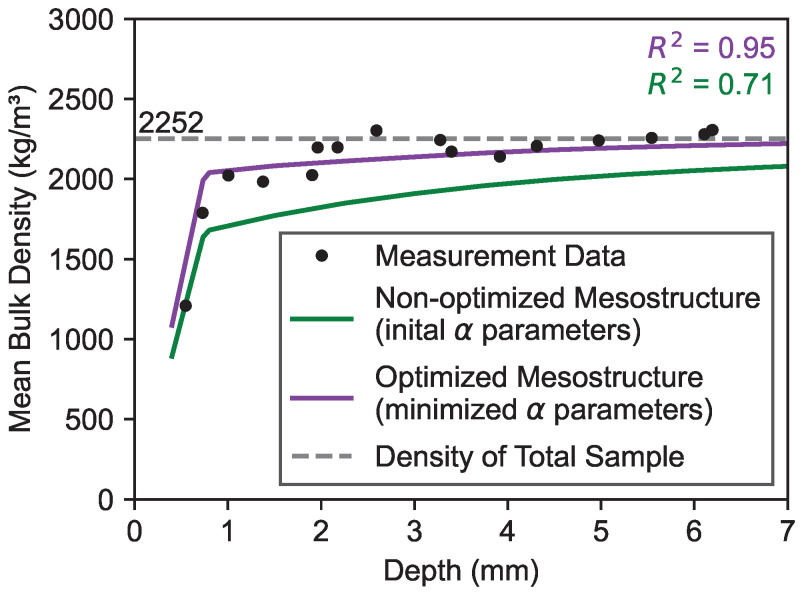
Comparison of mean bulk densities derived from measurement data and generated data.

Figure 14 shows the comparison of the depth-dependent mean bulk density changes of the measurement data and the generated mesostructures (initial and optimized α parameters). It can be seen that the initial distribution contains lower density values towards the near-surface region of the specimen compared to the measurement data ($R^{2}=0.71$). After the optimization process, which causes a migration of smaller particles towards the near-surface regions, the density values of the generated mesostructure increases and overlaps with the measurement data ($R^{2}=0.95$). With increasing depth, the mean bulk density values of both the optimized mesostructure and measurement data converge towards the total density of the sample (2252 kg/m^3^).

## 4. Comparison of Volume Fractions

**Figure 15:**
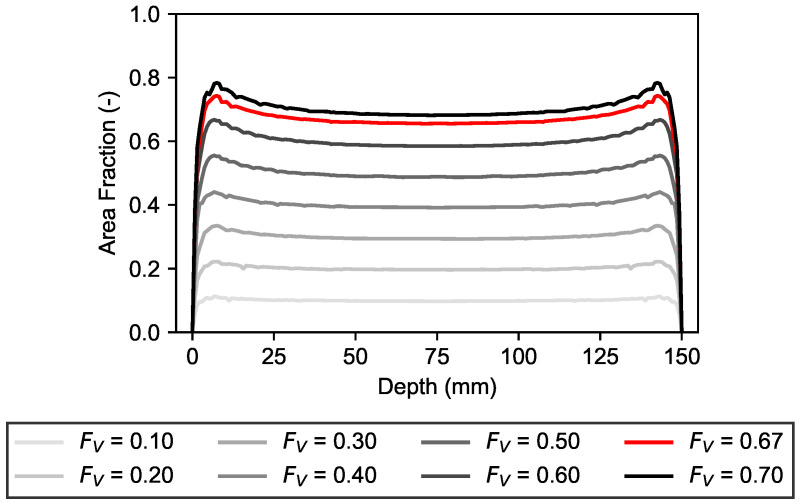
Comparison of aggregate area fractions $F_{A}$ for increasing volume fractions $F_{V}$.

The grading curve of Section 3.2 has been used to generate aggregates for different volume fractions $F_{V}$. Afterwards, the resulting aggregates were used to generate different mesostructures and ultimately derive the depth-dependent area fractions $F_{A}$. Figure 15 shows the resulting area fractions $F_{A}$ for volume fractions $F_{V}$ ranging from 0.10 to 0.70. These values have been chosen purely to visualize the effect of increased aggregate volume fractions on the resulting area fractions. The volume fraction of $F_{V}$ = 0.67 of the concrete sample used in the experimental validation is visualized as a red line. It can be observed that, with increasing $F_{V}$, the migration of smaller particles also rises and causes an increasing $F_{A}$ in the near-surface regions of the specimen. It is important to note that the peak values of $F_{V}$ and thus $F_{A}$ are limited by the maximum packing density of the specific grading curve. It is the subject of future research to implement the functionality of limiting the generation to local $F_{V}$ values based on the maximum packing density. For comparison, random close packing fractions of polydisperse circles in a two-dimensional space have been reported between 0.84 and 0.93, depending on the investigated grading curves [26,27]. These values provide a physical plausibility constraint for the generated mesostructures.

## 5. Discussion

### 5.1. Geometric Origin of the Near-Surface Densification

The proposed distribution-optimized mesostructure estimation method enforces a linear cumulative aggregate volume function along the specimen depth, which corresponds to a constant area fraction. Due to the spherical shape of the aggregates, their individual cumulative volume contributions follow sigmoidal curves. Near the specimen boundaries, only a portion of the spherical volume contributes to the cumulative volume within the considered depth interval. As a result, the effective volume contribution per unit depth is reduced close to the boundaries. To compensate for this geometric effect, the optimization algorithm shifts smaller aggregates toward the boundary regions. This leads to an increased local area fraction and therefore to higher mean bulk density values in the near-surface zone. Importantly, this behavior results from geometric constraints and is not imposed by predefined segregation rules. The method therefore captures the geometric component of the wall effect, i.e., the modification of aggregate distributions caused by boundary-induced geometric constraints on particle contributions [11,19,20]. It does not explicitly model rheological processes during casting, gravitational segregation, bleeding, or vibration effects. Instead, it isolates the structural consequences of geometric confinement under the assumption of statistical homogeneity in the bulk material. Such boundary-sensitive mesostructure indicators may also support monitoring-based assessments of structural materials where localized heterogeneity affects performance [28,29]. In addition, near-surface densification may influence transport properties and thus affect durability-related processes such as freeze-thaw damage or chloride ingress [25,30,31]. The presented framework may therefore provide useful boundary-condition descriptors for future durability simulations. Furthermore, the ability to incorporate non-spherical aggregate shapes should be considered, as aggregate shape and surface significantly influence concrete parameters [32].

### 5.2. Interpretation of the Experimental Agreement

The optimized mesostructure shows strong agreement with the experimentally determined depth-dependent mean bulk density ($R^{2}=0.95$). In contrast, the non-optimized configuration underestimates the near-surface density ($R^{2}=0.71$), indicating that a uniform aggregate depth distribution cannot reproduce the measured profile. The improved agreement suggests that geometric boundary effects contribute to the observed density increase in the near-surface region of the investigated specimen [33,34]. However, the experimental validation was performed for only one concrete mixture and one specimen geometry. Although the numerical investigations for different volume fractions show a consistent trend of increasing near-surface fine aggregate accumulation with increasing aggregate content, further experimental studies are required to confirm the general applicability of the method for different grading curves, maximum aggregate sizes, and casting conditions. Future work should consider coupling the approach with experimentally resolved mesostructures; for example, the use of imaging techniques such as X-ray computed tomography.

### 5.3. Symmetry Assumption of Boundary Conditions

The use of symmetric Beta distributions (β=α) assumes identical boundary conditions at opposing specimen faces [11,19,20]. In the experimental configuration, the specimen was cast in a cubic mold with comparable formwork conditions, which supports the assumption of symmetric boundary conditions between the vertical faces of the sample, i.e., along the x-axis or y-axis, see Figure 1a. In practical applications, casting direction, bleeding, sedimentation, or surface finishing may introduce asymmetric boundary effects [35,36], specifically along the vertical sample direction, i.e., the z-axis, see Figure 1a. The presented framework can be extended by allowing β≠α, which would enable asymmetric depth distributions when required. Such an extension would allow the method to account for directional casting effects or asymmetrical phenomena.

### 5.4. Limitation Regarding Maximum Packing Density

The current implementation does not explicitly include a local maximum packing constraint. Although the global aggregate volume fraction remains within realistic limits, local area fractions near the boundaries may approach values that should be verified against established packing density models [37,38]. Future work should therefore include local packing constraints to ensure the physical legitimacy of generated mesostructures, particularly for high aggregate volume fractions. Including such constraints would further improve the physical consistency of the model.

## 6. Conclusions

A novel methodology has been presented, which generates concrete mesostructures by minimizing the $R^{2}$ score between the actual and idealized homogeneous cumulative volume fraction functions, $F_{V}\left(x\right)$, of a polydisperse distribution of spherical aggregates. Ensuring the assumption of a constant $F_{A}\left(x\right)$ and therefore linear $F_{V}\left(x\right)$ within the geometry, the aggregates in the system are assembled in respect to the physical boundaries of the formwork. This results in smaller particles migrating towards the near-surface regions of the sample as a consequence of the optimization process. The presented method isolates and models the geometric contribution of boundary-induced aggregate redistribution. While it does not explicitly simulate casting-induced segregation mechanisms, it provides a framework for incorporating wall effects into mesoscale concrete simulations.

For systems with high amounts of individual aggregates, the presented method assumes groups of equal aggregate sizes (based on grading curve). The distributions of the grain size groups are described by a symmetrical Beta function. As a result, each grain size group can be described with one parameter α. The current iteration of the method only considers spherical aggregates. The spherical representation was chosen to enable the analytical derivation of cumulative volume functions and efficient optimization. For a more realistic representation of the aggregates, a different method for the generation of the aggregates has to be chosen which is able to numerically evaluate the cumulative volume contributions of non-spherical aggregates. The cumulative-volume-based optimization principle itself is not limited to spherical particles. However, during the generation of the mesostructure, the segmentation of grain size groups and representation of the aggregates with Beta distributions is not possible for unique, non-spherical aggregates. In this case, the individual positions of the aggregates need to be minimized. In addition, a rotation angle can be added to the optimization process to account for different aggregate rotations.

Generated mesostructures were compared with experimental data. Direct observation of three-dimensional aggregate distributions would require destructive sectioning or X-ray computed tomography, which was beyond the scope of this study. Hence, for this study, the mean bulk density of a concrete sample was determined both experimentally and virtually. The mesostructure generated with the optimization method yielded good agreement with the measurement data ($R^{2}=0.95$). This can be attributed to the migration of smaller particles towards the near-surface regions of the sample, which increased the density of the border zone. The density of a uniform, non-minimized aggregate distribution resulted in lower values compared to the measurement data ($R^{2}=0.71$). The presented validation demonstrates the feasibility of the approach for the investigated specimen configuration.

Furthermore, the method assumes the densities of both aggregates and cement matrix as constant values. In future iterations, the density of the cement matrix should also be viewed as a depth-dependent function. Analogously, different density values for the individual grain size groups should be considered. In addition, air voids were not considered in the presented two-phase representation of the concrete mesostructure but may be incorporated as an additional phase in future extensions. The generation of mesostructures with different volume fractions $F_{V}$ shows an increasing accumulation of smaller aggregates towards the near-surface regions of the sample. Future research will focus on implementing local packing constraints, asymmetric boundary conditions, and extensions toward non-spherical aggregate geometries.

## Figures and Tables

**Table 1:** Grain size distribution.

**Mesh size (mm)**	0.063	0.125	0.25	0.5	1	2	4	8	16
**Passing rate (wt%)**	0.17	0.36	7.39	18.13	29.74	49.11	81.1	98.2	100

**Table 2:** Concrete mixture proportions.

Cement (-)	w/c (-)	Cement (kg/m^3^)	Water (kg/m^3^)	Aggregates (kg/m^3^)	Volume Fraction $F_{V}$ (-)
CEM I	0.55	362	199.1	1759	0.67

**Table 3:** Number of aggregates for each grain size group.

**Grain size group (mm)**	0.125	0.25	0.5	1	2	4	8
**Number of aggregates (−)**	4,201,204	19,430,557	3,710,601	501,398	104,566	21,587	1443

**Figure A1:**
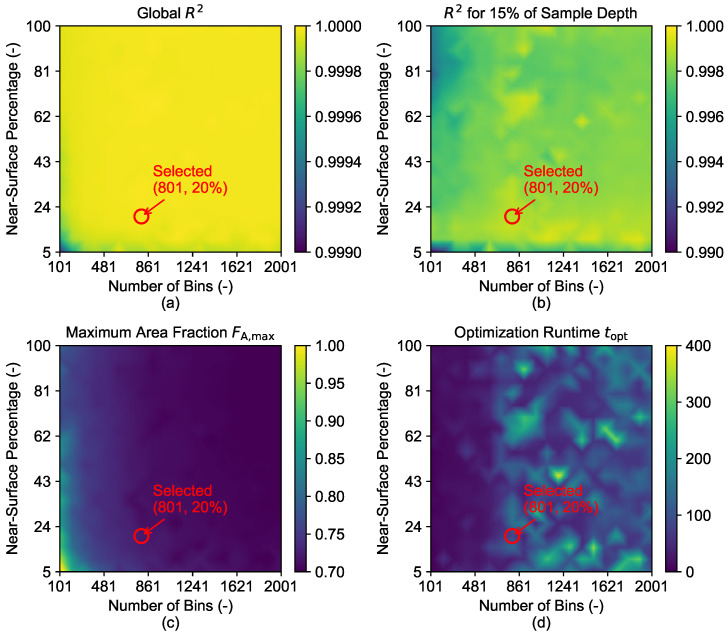
Results of the parametric sensitivity analysis. (**a**) Global $R^{2}$; (**b**) $R^{2}$ for 15% of sample depth; (**c**) maximum local area fraction $F_{A,\max}$; (**d**) optimization runtime $t_{\mathrm{opt}}$. The red marker indicates the parameter combination selected for the simulations presented in this study.

## Data Availability

The original contributions presented in this study are included in the article. Further inquiries can be directed to the corresponding author.

## Appendix A. Parametric Sensitivity Analysis of Optimization Parameters

A parametric sensitivity analysis of the optimization parameters was conducted to assess the influence of the number of discretization bins $n_{\mathrm{bins}}$ and the near-surface percentage $p_{\mathrm{ns}}$ used in the optimization objective. The parameter $n_{\mathrm{bins}}$ controls the discretization of the specimen depth used for evaluating the cumulative volume function. The parameter $p_{\mathrm{ns}}$ specifies the fraction of the specimen depth included in the loss function. Figure A1 shows heatmaps of the global coefficient of determination, the near-surface coefficient of determination evaluated within the first 15% of the specimen depth, the maximum local area fraction $F_{A,\max}$, and the optimization runtime $t_{\mathrm{opt}}$ of each parameter combination.

The results indicate that the global fit remains consistently high ($R^{2}$ > 0.999) across a wide parameter range, demonstrating the robustness of the optimization formulation. The lowest $R^{2}$ scores can be identified at both low $n_{\mathrm{bins}}$ and small $p_{\mathrm{ns}}$. In contrast, the near-surface accuracy shows a slightly more pronounced dependence on the near-surface percentage used during optimization. Very small $p_{\mathrm{ns}}$ values (<15%) tend to reduce the resulting $R^{2}$ scores, whereas values between approximately 20% and 75% yield consistently high near-surface agreement. A combination of low $n_{\mathrm{bins}}$ and $p_{\mathrm{ns}}$ > 75% results in a decrease in $R^{2}$ scores. Furthermore, the results indicate that $n_{\mathrm{bins}}$ primarily controls the numerical resolution of the optimization problem. Increasing the bin count improves the agreement between the generated and target grading curves, but the improvement becomes marginal beyond approximately 800 bins.

The maximum local area fraction $F_{A,\max}$ increases with both the number of bins and the near-surface percentage, reflecting the increased spatial flexibility of the optimization. For low $n_{\mathrm{bins}}$ and small $p_{\mathrm{ns}}$, local area fractions approach values close to the theoretical packing limit of polydisperse circular packings. As discussed in Section 4, random close packing fractions of polydisperse circles in a two-dimensional space have been reported between 0.84 and 0.93, depending on the investigated grading curves [26,27]. These values provide a physical plausibility constraint for the generated mesostructures.

The computational cost of the optimization increases primarily with the number of discretization bins. In contrast, the influence of the near-surface percentage on the runtime is comparatively small. However, increased runtime spikes were recorded for small $p_{\mathrm{ns}}$ < 20%, as well as for $p_{\mathrm{ns}}$ > 60%. Overall, the behavior of the optimization runtime $t_{\mathrm{opt}}$ reflects the increased resolution of the cumulative volume function with larger bin counts.

Based on these observations, primarily in regards to the values of $F_{A,\max}$ and $t_{\mathrm{opt}}$, a parameter combination of $n_{\mathrm{bins}}$ = 801 and $p_{\mathrm{ns}}$ = 20% was selected for the simulations presented in this study, representing a compromise between numerical accuracy, near-surface agreement, physical plausibility, and computational efficiency.
